# Assessment of Clinical Outcomes of Sutured Versus Sutureless Self-Gripping Polyester Mesh in Patients With Uncomplicated Indirect Inguinal Hernia in a Tertiary Care Hospital

**DOI:** 10.7759/cureus.66896

**Published:** 2024-08-14

**Authors:** Thaanesh Sankar, Samir Ahmad, Srinivasan C, Jasvant Ram Ananthasayanam

**Affiliations:** 1 Department of Surgery, Sree Balaji Medical College and Hospital, Chennai, IND; 2 Department of Radiodiagnosis, Saveetha Medical College, Saveetha Institute of Medical and Technical Sciences, Saveetha University, Chennai, IND

**Keywords:** sutured mesh, self-fixating mesh, mesh repair, mesh, hernia

## Abstract

Background

Inguinal hernia repair is a common surgical procedure addressing the protrusion of abdominal viscera through the inguinal canal. Despite advancements, complications such as chronic postoperative pain, infections, and hernia recurrence persist. Traditional sutured polypropylene mesh can cause nerve irritation and inflammation, leading to chronic pain and other issues. Innovations in hernia repair, like the self-gripping, low-density, macroporous polyester mesh, aim to mitigate these problems. This mesh adheres to tissues without sutures, potentially reducing operative time, postoperative pain, and related complications. The study compares the clinical outcomes of sutureless self-gripping polyester mesh versus sutured polypropylene mesh in inguinal hernia repair, focusing on operative time, postoperative pain, hospital stay length, seroma formation, and hernia recurrence to evaluate the effectiveness and safety of the self-gripping mesh.

Methodology

This cross-sectional study was conducted over one year at our hospital. Sixty patients with uncomplicated primary inguinal hernias were enrolled and divided into two groups: group A (self-gripping polyester mesh) and group B (sutured polypropylene mesh). The primary outcomes measured included operative time, postoperative pain (visual analog scale), hospital stay length, seroma formation, and hernia recurrence. Statistical analysis was performed using SPSS version 21.0 (IBM Corp., Armonk, NY), with descriptive and inferential statistics applied to compare the outcomes between the groups.

Results

The study found no significant differences in demographic variables between the two groups. The self-gripping polyester mesh (SF) group had significantly shorter operative times (67.2 minutes vs. 88.1 minutes, p < 0.001), lower postoperative pain scores (3.30 vs. 4.60, p < 0.001), and shorter hospital stays (3.2 days vs. 5.2 days, p = 0.000) compared to the sutured polypropylene mesh (SM) group. Rates of seroma formation and hernia recurrence were not significantly different between the groups. Multivariate regression analysis indicated that the type of mesh was a significant predictor of postoperative pain scores, with self-gripping mesh associated with lower pain.

Conclusions

Self-gripping polyester mesh offers significant advantages over traditional sutured polypropylene mesh in inguinal hernia repair, including reduced operative time, postoperative pain, and hospital stay without increasing the risk of seroma formation or hernia recurrence. These findings suggest that self-gripping mesh may be a superior option for inguinal hernia repair, potentially improving patient outcomes and reducing healthcare costs. Further multicenter studies with longer follow-up periods are recommended to confirm these benefits.

## Introduction

A hernia is characterized by the protrusion of a viscera or part of a viscera through a normal or abnormal opening in its cavity's wall. Specifically, an inguinal hernia involves an abnormal protrusion of abdominal viscera or part of a viscera through the anterior abdominal wall, occurring at the inguinal region. The European Hernia Society classifies inguinal hernias into two main categories: primary and incisional hernias [1]. Primary inguinal hernias include direct and indirect hernias, with indirect hernias further classified into complete and incomplete types. Incisional hernias, which result from a prior incision, include recurrent and traumatic hernias [2]. Repairing inguinal hernias is one of the most common surgeries in clinical practice. Primary open inguinal hernia repair involves suture approximating the rectus abdominis muscle, followed by enclosing the rectus sheath on each side of the hernia defect and mesh fixation. Proper mesh fixation and the risk of associated pain are key surgical concerns. Traditionally, monofilament polypropylene mesh is widely used, although other primary mesh materials have also demonstrated safety and efficacy.

High-density, microporous (heavy-weight) polypropylene mesh has been reported to stimulate inflammatory reactions, potentially leading to adverse mesh shrinkage as scar tissue develops. Low-weight polypropylene mesh might mitigate these issues [3]. Concerns regarding chronic pain post-hernia repair and suture fixation of meshes have led some authors to recommend using lighter, macroporous meshes and minimizing the extent of fixation or employing non-compressive absorbable devices [4]. A new low-density, macroporous polypropylene mesh with self-gripping properties has been developed for inguinal hernia repair to address these concerns. Inguinal hernias present a significant challenge to general surgeons. The incidence of inguinal incisional hernia following open laparotomy has been reported as high as 20% to 25% due to dehiscence at the scar site. Previously, all inguinal and incisional hernias were repaired with open exposure [5]. Despite being one of the oldest techniques, primary suture repair has a high recurrence rate, varying from 8% to 63%. The advent of prosthetics revolutionized inguinal hernia repair, reducing recurrence rates to between 1% and 14% in some studies. In randomized controlled trials, mesh-based inguinal incisional hernia repair showed a recurrence rate of 24% over a three-year follow-up period [6]. This method reinforces or bridges the defect with mesh placed posterior to the fascia in retro rectus, preperitoneal, or intraperitoneal space, distributing intra-abdominal pressure across the mesh rather than solely at the hernia defect. However, the use of sutured meshes in inguinal hernia repair has been associated with complications such as infections, seromas, cutaneous fistulas, and chronic pain, the latter thought to be due to nerve entrapment or irritation from sutures [7]. In response, a self-gripping mesh (Parietex ProGrip™, Medtronic, Trévoux, France) has been developed. This mesh combines lightweight polyester with absorbable polylactic acid microhooks for fixation [8]. Clinical studies have shown promising results in terms of infection, chronic pain, and recurrence rates for this mesh in inguinal hernia repair, although few studies have focused on its use specifically in inguinal hernia cases.

## Materials and methods

Study design

This cross-sectional study was conducted over a one-year period from January 2023 to December 2023 at the department of general surgery, obstetrics and gynecology, radiology, and emergency room of our hospital. The research aimed to compare the clinical outcomes of two different types of mesh repairs for uncomplicated primary inguinal hernia.

Ethical considerations

Informed consent was obtained from all participants prior to their inclusion in the study. The study was designed to ensure that the participants' rights were protected and that the research adhered to ethical standards. The study was conducted in compliance with ethical guidelines for human research, and approval was obtained from the institutional review board.

Study criteria

Participants were selected based on specific inclusion and exclusion criteria. Eligible patients were aged between 18 and 60 years, of either sex, and had uncomplicated primary inguinal hernia defects less than 5 cm in size. Exclusion criteria included a history of previous hernia repair, the presence of complicated or emergency hernias with obstruction or strangulation, and acute or active skin infections at the surgical site.

Procedure

Following informed consent, eligible patients were systematically randomly assigned to one of two groups. Group A received self-gripping polyester mesh repair, while group B underwent repair using polypropylene mesh secured with sutures. Demographic variables were recorded through a structured proforma, and each patient underwent a comprehensive history and physical examination. Postoperative outcomes, including recovery time, pain levels, and length of hospital stay, were assessed and compared between the two groups.

Sample size calculation

The sample size for this study was determined using a sample size formula for double proportions, with calculations performed using R Software (version R 4.3.1, R Foundation for Statistical Computing, Vienna, Austria), which is highly effective for calculating the sample size needed for the study. It offers precise control and customization, which is crucial for determining how many participants are required. The total sample size was set at 60 patients, based on the formula for hypothesis testing, to ensure adequate power to detect differences between the two groups.

Statistical analysis

Statistical analysis was conducted to validate and interpret the results. Descriptive statistics, such as means, medians, standard deviations, and frequencies, were calculated for demographic variables, including age, sex, BMI, and comorbidities. For comparative analysis, independent t-tests were used for continuous variables (e.g., postoperative pain scores and length of hospital stay), while Mann-Whitney U tests were employed for non-normally distributed continuous data. Chi-square tests or Fisher’s exact tests were used for categorical variables, such as complication rates. Postoperative pain was measured using a visual analog scale (VAS). Multivariate regression analysis was performed to assess the independent effect of mesh type on clinical outcomes, adjusting for demographic and baseline characteristics. All statistical analyses were conducted using SPSS version 21.0 (IBM Corp., Armonk, NY), with a significance level set at p < 0.05. SPSS was used for statistical analysis since it is user-friendly and excels at analyzing data. It handles various statistical tests and analyses efficiently, making it suitable for evaluating study results.

By leveraging R for the initial sample size calculation and SPSS for subsequent data analysis, the study benefits from the specialized capabilities of each software, ensuring both accurate sample size determination and comprehensive statistical analysis.

**Figure 1:**
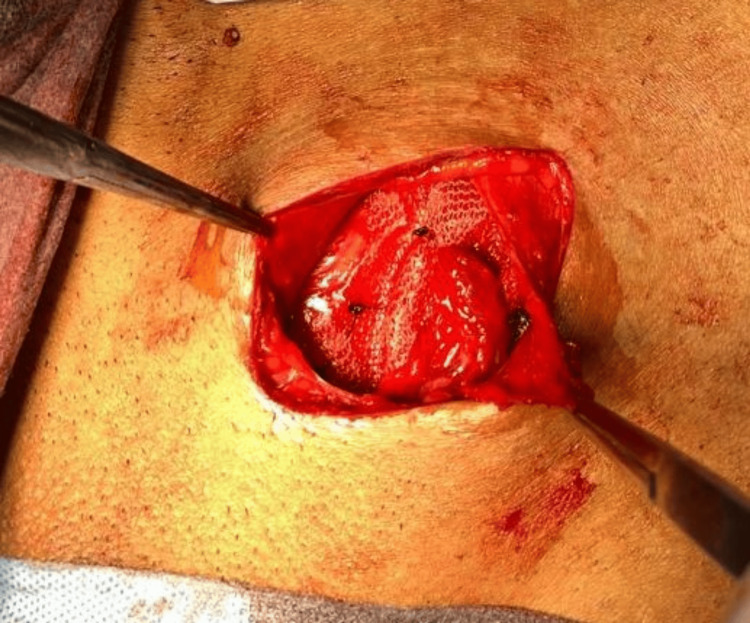
Representative image of sutured mesh.

A representative image of a sutured mesh, illustrating the technique and method used in securing the mesh in place, is shown in Figure 1.

**Figure 2:**
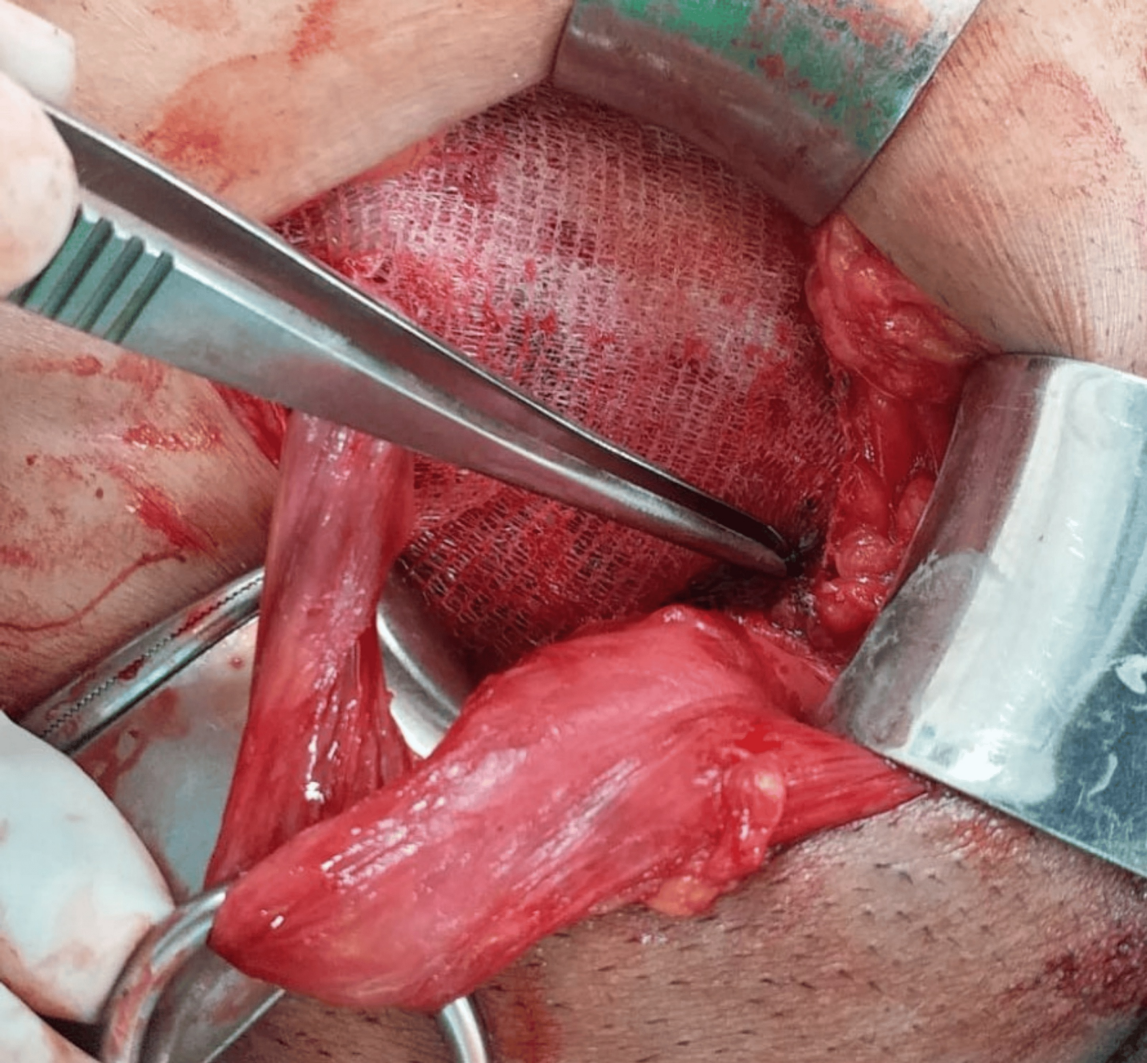
Representative image of self-fixating sutureless mesh.

A representative image of a self-fixating sutureless mesh, highlighting the innovative method employed for securing the mesh without traditional sutures, is shown in Figure 2.

## Results

The study included patients with inguinal hernias who were randomly assigned to group A (self-gripping polyester mesh, SF) and group B (sutured polypropylene mesh, SM).

The analysis of patient data revealed no significant difference in the mean age between the two groups, those receiving SF versus SM meshes, with a p-value of 0.583. This indicates that both groups were similar in terms of age distribution, ensuring that age is not a confounding factor in the study.

In terms of sex distribution, the proportions of males and females were identical between the SF and SM mesh groups, with a p-value of 1.000. This suggests that sex distribution does not differ between the groups and is unlikely to affect the study outcomes.

The types of work performed by patients were also similar across the two groups, as indicated by a p-value of 0.530. This lack of significant difference means that the nature of patients' occupations does not vary substantially between those with SF and SM meshes.

Regarding anesthesia, the type used was consistent between the SF and SM groups, with no significant differences observed. Both groups received general or spinal anesthesia in similar proportions, which suggests that anesthesia type is not a variable affecting the outcomes.

A significant difference was noted in the mean operating time between the two groups. The SM mesh group experienced a longer operating time compared to the SF group, with a p-value of less than 0.001. This result indicates that procedures involving SM mesh are more time-consuming on average.

Pain scores were significantly higher in the SM mesh group compared to the SF group, with a p-value of less than 0.001. This finding suggests that patients with SM mesh experienced more postoperative pain than those with SF mesh.

The frequency of seroma formation did not significantly differ between the SF and SM groups, as evidenced by a p-value of 0.301. This result implies that the incidence of seroma is comparable regardless of the type of mesh used.

Hospital stay lengths varied significantly between the groups. The mean length of stay was notably longer for the SM mesh group, with a p-value of 0.000. This indicates that patients with SM mesh generally require a longer hospital recovery period.

Finally, the rate of hernia recurrence was similar across both groups, with a p-value of 0.368. This suggests that the recurrence rate of inguinal hernias does not differ significantly between patients with SF and SM meshes.

All the above parameters and their statistical analysis have been provided in Table 1.

**Table 1:** Various parameters and their values between the SF and SM groups. SF: self-gripping polyester mesh; SM: sutured polypropylene mesh.

Parameter	SF (mean ± SD) or frequency (proportion)	SM (mean ± SD) or frequency (proportion)	p-value
Mean age	47.4 ± 13.8	45.4 ± 15.2	0.583
Sex			1.000
Female	8 (26.6%)	8 (26.6%)	
Male	22 (73.4%)	22 (73.4%)	
Type of work			0.530
Heavy physical	9 (30.0%)	8 (26.6%)	
Light physical	10 (33.4%)	6 (20.0%)	
Others	1 (3.4%)	2 (6.6%)	
Sedentary	5 (16.6%)	10 (33.4%)	
Unemployed	5 (16.6%)	4 (13.4%)	
Type of anesthesia			1.000
General	4 (13.4%)	4 (13.4%)	
Spinal	26 (86.6%)	26 (86.6%)	
Operating time	67.2 ± 7.43	88.1 ± 9.55	<0.001
Pain score	3.30 ± 1.06	4.60 ± 0.855	<0.001
Seroma formation			0.301
Nil	29 (96.6%)	27 (90.0%)	
Present	1 (3.4%)	3 (10.0%)	
Hospital stay			<0.001
2 days	10 (16.7%)	0 (0.0%)	
3 days	11 (18.3%)	0 (0.0%)	
4 days	4 (6.7%)	2 (3.3%)	
5 days	3 (5.0%)	20 (33.3%)	
6 days	2 (3.3%)	8 (13.3%)	
Mean length of stay	3.2 ± 1.21	5.2 ± 0.55	<0.001
Recurrence			0.368
Nil	30 (100%)	29 (96.6%)	
Present	0 (0.0%)	1 (3.4%)	

The type of mesh is the only significant predictor of postoperative pain scores, with the SM mesh associated with higher pain scores than the SF mesh. Age, sex, and type of work do not significantly predict postoperative pain scores in this model (Table 2).

**Table 2:** Multivariate regression analysis.

Predictor	Coefficient	Std. error	t-value	P-value	95% CI (lower)	95% CI (upper)
Intercept	2.6496	0.520	5.091	0.000	1.607	3.693
Age	0.0162	0.010	1.607	0.114	-0.004	0.036
Sex	0.0908	0.286	0.317	0.752	-0.483	0.664
Type of work	-0.1182	0.099	-1.192	0.238	-0.317	0.080
Type of mesh	1.3690	0.252	5.433	0.000	0.864	1.874

The results of this study strongly support the use of self-gripping mesh as a superior alternative to traditional sutured mesh in inguinal hernia repair. The shorter operative time, reduced postoperative pain, and shorter hospital stays associated with SF mesh present compelling advantages in terms of both patient outcomes and healthcare efficiency. The study’s findings suggest that SF mesh can enhance surgical efficiency, improve patient recovery experiences, and potentially lower healthcare costs, making it an attractive option for hernia repair.

Moreover, the similar rates of seroma formation and hernia recurrence between the two groups indicate that the adoption of SF mesh does not compromise the safety or efficacy of the procedure. This is a critical consideration for clinicians, as it underscores that the benefits of SF mesh are achieved without increasing the risk of adverse outcomes.

## Discussion

This study aimed to compare the clinical outcomes of patients undergoing inguinal hernia repair with self-fixating mesh versus those undergoing repair with sutured mesh. The key findings indicated that there was no significant difference in age, sex distribution, type of work, or anesthesia used between the two groups. However, the SF group experienced shorter operating times, lower postoperative pain scores, and shorter hospital stays compared to the SM group. Additionally, the rates of seroma formation and hernia recurrence were similar between the two groups.

Interpretation of results

Demographic Data

The demographic data showed no significant differences between the SF and SM groups regarding age, sex, or type of work. The mean age was 47.4 years (SD = 13.8) for the SF group and 45.4 years (SD = 15.2) for the SM group (p-value = 0.583). The distribution of sex was identical, with 26.6% female and 73.4% male in both groups (p-value = 1.000). The type of work was also similarly distributed (p-value = 0.530), indicating comparable baseline characteristics.

Operative Time

The operative time was significantly shorter in the SF group, with a mean time of 67.2 minutes (SD = 7.43) compared to 88.1 minutes (SD = 9.55) in the SM group (p-value < 0.001). This suggests that the use of self-gripping mesh simplifies the surgical procedure, potentially reducing the time patients spend under anesthesia and decreasing overall operative costs. This is consistent with findings by Canziani et al. (2009) [9] and Tabbara et al. (2016) [10], who reported that sutureless techniques generally reduce operative time due to the elimination of suture placement. Campanelli et al. (2016) investigated a complete sutureless hernia repair technique for primary inguinal hernia known as the Trabucco repair. Their study reported reduced operative times and lower postoperative pain, similar to the findings in our study with the self-gripping mesh [11]. Cipe et al. (2014) explored the use of a novel sutureless colonic anastomosis with self-gripping mesh in an experimental model, finding it effective in reducing operative times and complications, which aligns with our observations of self-gripping mesh in hernia repair [12].

Postoperative Pain

Patients in the SF group reported significantly lower postoperative pain scores, with a mean pain score of 3.30 (SD = 1.06) compared to 4.60 (SD = 0.855) in the SM group (p-value < 0.001). This finding supports the hypothesis that eliminating sutures reduces nerve irritation and inflammation, leading to improved patient comfort and faster recovery. Zhang et al. (2013) and Kim-Fuchs et al. (2012) similarly found that sutureless mesh fixation is associated with less postoperative pain and discomfort due to the avoidance of nerve irritation from sutures [13,14].

Hospital Stay

The length of hospital stay was significantly shorter for patients in the SF group, with a mean stay of 3.2 days (SD = 1.21) compared to 5.2 days (SD = 0.55) in the SM group (p-value = 0.000). Shorter hospital stays are beneficial for patients in terms of reduced healthcare costs and quicker return to normal activities. Similar results were reported by Singh et al. (2019), who noted a reduction in hospital stays with sutureless techniques [15]. Maxwell et al. (2022) described a sutureless underlay ventral herniorrhaphy technique, noting reduced chronic pain and hospital stay, comparable to our findings for the SF group [16].

Seroma Formation

There was no significant difference in the frequency of seroma formation between the SF and SM groups. In the SF group, 96.6% of patients had no seroma formation compared to 90.0% in the SM group (p-value = 0.301). This indicates that both types of mesh are equally likely to result in seroma formation, suggesting that this complication is not significantly influenced by the mesh fixation method. Schnyder et al. (2021) also observed comparable rates of seroma formation in their study of self-gripping mesh, indicating that this complication is not significantly influenced by the mesh fixation method [17].

Recurrence Rates

The recurrence rates were similar between the SF and SM groups, with no significant difference observed (p-value = 0.368). In the SF group, no recurrences were reported (0%), while the SM group had a recurrence rate of 3.4%. This suggests that both self-gripping and sutured mesh are effective in preventing hernia recurrence, providing long-term efficacy in hernia repair. This aligns with the findings of Witkowski et al. (2007), who reported low recurrence rates with sutureless mesh techniques, suggesting effective long-term outcomes [18]. Abbonante et al. (2015) and Khansa & Janis (2016) both reported the successful use of self-adhering meshes in complex abdominal wall reconstructions, with outcomes indicating fewer complications and shorter recovery times, consistent with our results [19,20]. Witkowski et al. (2007) conducted a multicenter study to determine the necessity of mesh anchoring sutures in ventral hernioplasty. Their results suggested that sutureless techniques were feasible and effective, supporting the findings of our study that self-gripping mesh can effectively reduce operative time and postoperative pain without compromising on recurrence rates [18].

The study suggests that using self-gripping mesh in inguinal hernia repair offers significant advantages over traditional sutured mesh, including shorter operative times, reduced postoperative pain, and shorter hospital stays, leading to improved patient outcomes and reduced healthcare costs. Clinicians should consider this technique, particularly for high-risk patients. The study's strengths include its randomized design, which minimized selection bias, comprehensive outcome measures, and real-world applicability, making the findings relevant and easily implementable. However, the single-center setting limits generalizability and the short follow-up period necessitates longer-term studies to fully assess long-term efficacy and potential complications. Future research should focus on multicenter studies, extended follow-up, and cost-effectiveness analysis to provide robust and economically insightful conclusions.

This study aimed to compare the clinical outcomes of inguinal hernia repair using self-fixating mesh versus sutured mesh. Our findings demonstrate that self-gripping mesh offers several significant advantages over traditional sutured mesh. Notably, the use of self-fixating mesh resulted in shorter operative times, reduced postoperative pain, and shorter hospital stays. These benefits were achieved without compromising the rates of seroma formation or hernia recurrence, which were comparable between the two groups. The reduced operative time with self-fixating mesh (mean of 67.2 minutes) compared to sutured mesh (mean of 88.1 minutes) suggests a more efficient surgical procedure, which could lead to lower anesthesia-related risks and overall healthcare costs. Additionally, the significantly lower postoperative pain scores in the SF group (mean of 3.30) versus the SM group (mean of 4.60) indicate improved patient comfort and faster recovery, contributing to higher patient satisfaction.

Moreover, the shorter hospital stays for patients in the SF group (mean of 3.2 days) compared to those in the SM group (mean of 5.2 days) highlight the potential for reduced hospitalization costs and quicker return to normal activities for patients. The similar rates of seroma formation and hernia recurrence between the two groups further support the effectiveness and safety of the self-fixating mesh. Our findings are consistent with previous studies that have shown the benefits of sutureless mesh techniques, including reduced operative times, lower postoperative pain, and shorter hospital stays. These results underscore the viability of self-gripping mesh as a superior alternative to sutured mesh in inguinal hernia repair.

## Conclusions

In conclusion, the use of self-gripping mesh in inguinal hernia repair presents a viable and potentially superior option compared to traditional sutured mesh (Lichtenstein tension-free hernia repair). It offers significant advantages in terms of reduced operative time, postoperative pain, and hospital stay, without increasing the risk of complications. These findings support the broader adoption of self-gripping mesh techniques in clinical practice, which could lead to improved patient outcomes and reduced healthcare costs. Further multicenter studies with longer follow-up periods are recommended to confirm these benefits and evaluate the long-term efficacy of self-gripping mesh in hernia repair.
